# Editorial for the Special Issue From Nanoinformatics to Nanomaterials Risk Assessment and Governance

**DOI:** 10.3390/nano11010121

**Published:** 2021-01-07

**Authors:** Iseult Lynch, Antreas Afantitis, Dario Greco, Maria Dusinska, Miguel A. Banares, Georgia Melagraki

**Affiliations:** 1 School of Geography, Earth & Environmental Sciences, University of Birmingham, Edgbaston, Birmingham B15 2TT, UK; 2 Department of Cheminformatics, NovaMechanics Ltd., Nicosia 1065, Cyprus; afantitis@novamechanics.com (A.A.); melagraki@novamechanics.com (G.M.); 3 Faculty of Medicine and Health Technology, Tampere University, 33100 Tampere, Finland; dario.greco@tuni.fi; 4 Environmental Chemistry Department, Norwegian Institute for Air Research, 2027 Kjeller, Norway; dusinska@nilu.no; 5 Institute for Catalysis, ICP-CSIC, Marie Curie 2, E-28049 Madrid, Spain; miguel.banares@csic.es

Ensuring the safe and responsible use of nanotechnologies and nanoscale materials is imperative to maximize consumer confidence and drive commercialization of nano-enabled products that underpin innovation and advances in every industrial sector. The enormous diversity of nanomaterials and their applications requires new approaches to governance, new approaches to managing the wealth of data relating to nanomaterials and their impacts on humans and the environment, and new approaches to risk assessment. Indeed, the regulatory landscape is evolving to account for the complexity and diversity of nanomaterials, including the recent introduction by the European Chemicals Agency of the concept of “nanoforms”, to distinguish individual members of a family of nanomaterials with a common core composition (e.g., carbon-family materials, TiO_2_ materials, etc.) but differing in size, shape, surface composition, or coating (for example), and “sets of nanoforms”, to group nanoforms that induce similar toxicological effects, so that a single set of characterization and toxicity data will cover the full set of nanoforms [1].

Informatics approaches for nanosafety assessment, including prediction of nanomaterials properties, interactions with biomolecules, cells and organisms, nanomaterials transformations, and biological effects and impacts of nanomaterials are contributing to data gap-filling, predictive modeling, and in silico tools for the risk assessment of nanomaterials. Key to facilitating progress in development of safe and sustainable nanomaterials applications, as well as risk governance and nanoinformatics approaches, is that high-quality data are available for re-use and fit for the purpose of developing predictive models, i.e., that are compliant with the FAIR (Findable Accessible, Re-usable, and Interoperable) principles. However, achieving this has been challenging, and is one of the themes addressed in the current Special Issue.

This Special Issue collects the most recent advances in governance of nanomaterials and in the emerging field of nanoinformatics. The 16 articles in this collection address three interlinked aspects of the topic: (1) data-management tools and approaches to make nanosafety data FAIR; (2) in silico tools for nanosafety assessment; and (3) experimental best practice in terms of data collection, preprocessing, and interrogation, to understand the mechanisms of action of nanomaterials, as shown schematically in Figure 1.

A range of tools to increase the FAIRness of nanosafety data have been developed, targeted towards experimental researchers who are the data generators, as well as focusing on the databases themselves. For example, Kochev et al. present a workflow to convert the spreadsheets beloved by experimentalists into a FAIR database via the NMDataParser tool (Version 1.1.4, https://github.com/enanomapper/nmdataparser), which was developed to streamline the mapping of the original file layout into the eNanoMapper semantic data model [2]. A key element of FAIR data is that they are re-usable, and the core feature of re-usability of data is that the dataset is fully described in terms of what the dataset is, how it was generated, what it contains, etc. These “data about the data” are called metadata, and what metadata are required for re-usability of nanosafety must be defined by the nanosafety community itself. Papadiamantis et al. present a community-driven metadata schema, introduce some “scientific FAIR principles” implementable by experimental researchers, and suggest that a new role, that of data shepherd, is required to support data management across the whole nanosafety data life cycle, from experimental design to deposition in publicly accessible databases [3]. Depositing data into a database is not a complete guarantee of FAIRness, as not all databases are created equal and there are very broad interpretations of the FAIR principles. Thus, the concept of maturity indicators for evaluation of dataset (database) FAIRness have emerged, and Ammar et al. present a reproducible computational workflow to assess data FAIRness in the life sciences, presenting the compliance with each of the principles as a FAIR balloon plot to summarize and compare dataset FAIRness [4]. On the basis of this analysis, recommendations for improvement of the databases include the use of standard schema for metadata and indexing of the database in registries of repositories that could increase FAIRness of datasets. An exciting example of the implementation of data management workflows into experimental practice is presented in Martinez et al., who investigated the toxicity of graphene-based materials to *Daphnia magna* alone and in combination with cadmium as a co-pollutant and explored the role of an acquired biomolecule corona on the graphene in mitigating the toxicity of cadmium [5]. The data were collected and captured by utilizing an Electronic Laboratory Notebook, and all the data were annotated with relevant ontology terms and integrated into the NanoCommons Knowledge Base (version 1.1; https://ssl.biomax.de/nanocommons/cgi/login_bioxm_portal.cgi), making the data interoperable with similar datasets and facilitating utilization of the experimental data in nanoinformatics workflows [5]. In a major step towards dataset interoperability, a community proposal for an extension of the InChI [6], a textual identifier for chemical substances that was designed to provide a standard way to encode molecular information and to facilitate the search for such information in databases (that is endorsed and supported by the International Union of Pure and Applied Chemistry (IUPAC)), to capture and encode the multi-component structures of nanomaterials in a machine-readable format, is presented by Lynch et al. [7]. Six case studies were conducted to elucidate the requirements for unambiguous description of nanomaterials and to demonstrate the utility of the nanomaterial InChI (NInChI) in linking disparate datasets, supporting determination of nanoforms and sets of nanoforms, and for nanoinformatics development and machine learning [6].

Exciting advances in predictive modeling of nanomaterials’ properties, their interactions with their surroundings, including biomolecules, and their impacts on cells are presented. Shateri et al. compare the performance of eight different machine-learning models for prediction of nanofluid viscosity against the predictions of the gold-standard model (the committee machine intelligent system, CMIS), using an experimental dataset consisting of 3144 data points of relative viscosity of 42 different nanofluid systems based on five features (temperature, the viscosity of the base fluid, nanoparticle volume fraction, size, and density) and found that all eight suggested models outperformed the baselines used in the literature, and five outperformed the CMIS [8]. An approach to generating the protein corona associated with nanomaterials in biological fluids from first principles, using free energy of adsorption, is described by Alsharif et al. [9]. A multiscale model of protein–nanomaterial interaction was generated by using adsorption energies of 59 human serum proteins and gold and titanium dioxide (anatase) nanoparticles, 2D and 3D protein descriptors, and statistical models for predicting the binding energy of proteins, enabling the rapid characterization of the affinity of nanomaterials for a wide range of proteins [9]. A robust and validated in silico model for prediction of metal oxide nanomaterial cytotoxicity (cell viability, measured as irreversible cell membrane damage) was developed, using a literature dataset on 24 metal oxides consisting of 15 physicochemical, structural, and assay-related descriptors and 62 atomistic computational descriptors, of which seven were identified as being significant for induction of cytotoxicity by Me_x_O_y_ nanomaterials [10]. This model was also provided with a user-friendly interface (Version 1.0, https://cellviability.cloud.nanosolveit.eu/), allowing users to apply it themselves, including tables of the computational descriptors for a range of metal oxide nanomaterials [10]. Two review papers complement the nanoinformatics advances. Utembe et al. review the state-of-the-art and emerging trends in Physiologically Based Pharmacokinetic (PBPK) modeling of nanomaterials, highlighting areas of progress, such as the inclusion of the mononuclear phagocyte system, and gaps that require further innovation, such as inclusion of organ- and species-specific nanomaterial corona formation, and nanomaterial dissolution, which is a key elimination process for some nanomaterials [11]. Furxhi et al. review the sequence of steps involved in implementing a machine-learning model, from data preprocessing to model implementation, model validation, and applicability domain determination; they also review the techniques and procedures of existing models that can be used readily to assemble new nanotoxicological in silico approaches [12].

Recognizing the growing importance of toxicogenomics analysis in providing mechanistic insights into nanomaterials modes of actions, and the hampering effect of poor study design and the lack of standardization in data generation and analysis, a series of three interlinked papers explore aspects of toxicogenomics, ranging from the experimental details (Part 1) to rigorous and reliable data preprocessing (Part II) and utilization of the toxicogenomics datasets in modeling (Part III), such as the development of adverse outcome pathways (AOPs). Part I provides guidelines on exposure time, dose and complex endpoint selection, sample quality considerations, and sample randomization [13]. Part II reviews the steps involved in transcriptomics data preprocessing, which spans multiple steps, including data quality checking, filtering, normalization, batch defect detection and correction, and defines the optimal tools and procedures to be employed to ensure the generation of homogeneous and unbiased data, allowing the development of more reliable, robust, and accurate predictive models [14]. Part III reviews the state-of-the-art of data modeling applied to transcriptomics data, including benchmark dose analysis, AOP modeling methodologies, network-based approaches to clarify mechanisms of action, and the emergence of Artificial Intelligence and deep learning (DL) approaches to enable more accurate chemical safety assessment [15]. Ede et al. review the current status and next steps for the development and use of the AOP framework in decision-making regarding the safety of nanomaterials, identifying opportunities and challenges for inclusion of AOPs into integrated approach to testing and assessing (IATA) strategies [16]. Meanwhile, Kohl et al. review the state-of-the-art in evaluation of genotoxicity, focusing on approaches for miniaturization, organ-on-a-chip and high-throughput methods, in standard human in vitro models, as well as new advanced 3D models that are closer to the in vivo situation [17]. Murphy et al. evaluate the economic efficacy of the widespread usage of nanomaterials coated textiles, which have antimicrobial properties and thus are proposed to reduce hospital-acquired infections (HAIs), utilizing an aggregated approach [18]. While this approach relies on some supposition, it allows for a comparison with similar data regarding standard treatments to reduce HAIs and provides a reasonable economic comparison. The analysis found that, relative to antiseptics, nanomaterial-coated textiles represent a significant clinical advantage, and also offer considerable cost savings [18].

In summary, this Special Issue of Nanomaterials collects a series of original research articles and review papers, providing new insights into the emerging state-of-the-art in nanosafety data management, for in silico hazard and risk assessment of nanomaterials, and in the application of these emerging approaches for decision-making and governance. It illustrates the breadth and diversity of the field and the range of innovative approaches being developed, to ensure the safe and responsible implementation of nanotechnologies. We are confident that this Special Issue will provide readers with an overview of the latest prospects in this rapidly evolving and cross-disciplinary field.

## Figures and Tables

**Figure 1:**
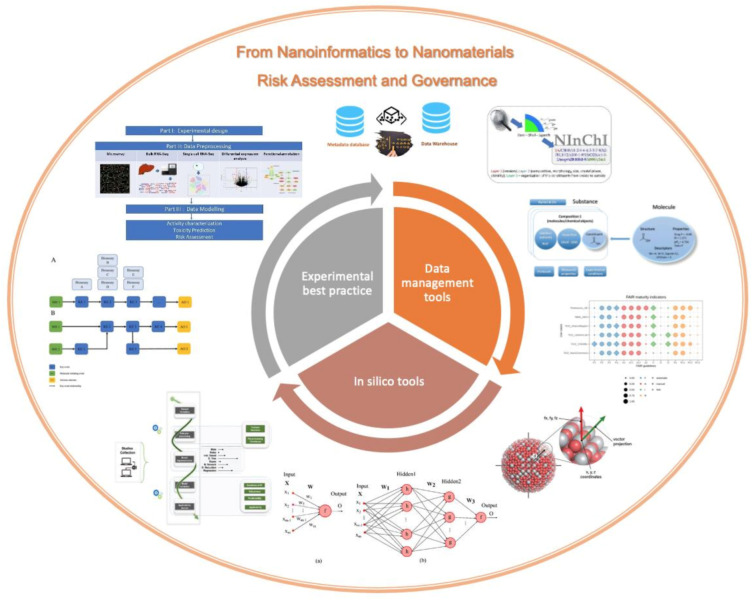
Schematic representation of the range of topics covered in the Special Issue and their clustering into three key areas, addressing (1) nanosafety data-management tools, (2) in silico tools for nanoinformatics and (3) experimental best practice to support data re-use for modeling and prediction of the impacts of nanomaterials on living systems. The smaller images around the central core are the graphical abstracts of a subset of the papers contained in the Special Issue.

## Data Availability

Not applicable.
